# AUV Navigation Correction Based on Automated Multibeam Tile Matching

**DOI:** 10.3390/s22030954

**Published:** 2022-01-26

**Authors:** Jochen Mohrmann, Jens Greinert

**Affiliations:** 1 DeepSea Monitoring/Marine Geosystems, GEOMAR Helmholtz Centre for Ocean Research Kiel, 24148 Kiel, Germany; 2 Institute of Geosciences, Christian-Albrechts University Kiel, Ludewig-Meyn-Str. 10-12, 24098 Kiel, Germany; jgreinert@geomar.de

**Keywords:** multibeam, bathymetric data, MBES, deep sea, terrain based navigation, AUV

## Abstract

Ocean science and hydroacoustic seafloor mapping rely on accurate navigation underwater. By exploiting terrain information provided by a multibeam echosounder system, it is possible to significantly improve map quality. This article presents an algorithm capable of improving map quality and accuracy by aligning consecutive pings to tiles that are matched pairwise. A globally consistent solution is calculated from these matches. The proposed method has the potential to be used online in addition to other navigation solutions, but is mainly targeted for post processing. The algorithm was tested using different parameter settings on an AUV and a ship-based dataset. The ship-based dataset is publicly available as a benchmark. The original accurate navigation serving as a ground truth, alongside trajectories that include an artificial drift, are available. This allows quantitative comparisons between algorithms and parameter settings.

## 1. Introduction

Most marine sciences rely on some kind of depth information of the ocean or sea and often use freely available datasets, such as GEBCO [1] or IBCAO [2], which are a compilation from satellite altimetry and ship-based hydroacoustic measurements using multibeam echosounder systems (MBES). For several geological and biological questions, high-resolution bathymetric data are crucial. Such questions range from detailed studies concerning location, spatial extent, and distribution of specific habitats, or the link to geochemical processes around hydrothermal vents in mid ocean ridge settings [3] or cold seeps at continental margins [4]. In addition, highly accurate bathymetric maps are needed for offshore construction and marine mineral resource exploration. In this respect, the assessment of Mn-nodules as potential ore resources in the abyss of the Clarion–Clipperton zone of the equatorial east Pacific is one area of great interest. Highly detailed bathymetric data, in conjunction with discrete sediment samples and photo surveys as ground truth, are used to accurately quantify the Mn-nodule density per square meter, with a few meters of resolution in 4500 m water depth [5].

Acquiring high-resolution data in water depth of 500 m or more has two major challenges: the angle between beams can physically not reduce much below $0.5^{\circ }$ for reasonably-sized transducers, which leads to a decrease in the across-track resolution, with depth. At the same time, the along-track resolution is reduced by a slower ping rate, due to an increased travel time of the signal. One solution is to bring the multibeam system closer to the seafloor on a stable platform, e.g., autonomous underwater vehicles (AUVs). With a speed of 3 to 4 kn and the possibility of tight turns, a common strategy is to perform predefined lawn-mowing patterns above the seafloor, keeping either water depth or altitude above the seafloor constant. Ship-based surveys can rely on real time global navigation satellite system (GNSS) information; using real time kinematic (RTK) correction near the shore allows having fixed error bounds with centimeter accuracy [6]. Due to attenuation of the electromagnetic signal, this is unfeasible underwater.

Most AUVs rely on an inertial measurement unit (IMU), motion sensors (accelerometers) and rotation sensors (gyroscopes). The inertial navigation system (INS) combines the readings and calculates the position, orientation, and velocity from these sensors using dead reckoning. Since there is no absolute reference and relative measurements are integrated, this method suffers from unbound error drifts in absolute positions that will increase with the traveled distance. In practice, this leads to drifts in the navigational data.

Technologies, such as ultra short baseline (USBL), allow one to indirectly access the GNSS positioning by using a surface vessel as a relay. The relative transformation between vessel and AUV is established by measuring the run time of an acoustic signal from the AUV towards multiple hydrophones on the vessel. USBL introduces additional errors [7]. The sound velocity within the water body is not constant and establishing the correct sound propagation direction can be faulty. This problem can be solved by positioning an array of hydrophones near the seafloor. The transponders of the long baseline system (LBL) need to be calibrated properly and have to be deployed in the vicinity of the mission area [8]. The deployment takes time and the area where good navigation can be achieved is limited. In practice, LBL position updates can lead to sudden positioning updates of the vehicle. Uncorrected, these jumps in the navigation cause unwanted artifacts, particularly in side scan data.

Inaccurate navigation becomes visible as inconsistency in depth maps between overlapping tracks. When such data are used for high-resolution gridding, artifacts in the form of wrong morphological features, such as ripples or steps, can be created. The results, especially when derivatives (such as slope or rugosity) are calculated to classify the surveyed regions, will create incorrect data and subsequent interpretations [9,10,11]. An uninformed algorithm will not be able to differentiate between artifacts and true morphology. A corrected navigation minimizes this problem; map quality, meaningfulness and validity will be improved.

An automatic correction of the AUV flight path can be achieved by using the multibeam data itself to improve the dead reckoning navigation. When conducting lawnmower patterns, adjacent survey lines will record the same area multiple times. The drift can be observed and used to retroactively correct the estimated flight path.

The need for automated renavigation of AUV multibeam flight paths in-situ on the AUV became prominent in the H2020 EU project ROBUST. The aim of this project was to develop AUV routines that allow for a complete autonomous multibeam mapping of the seafloor, the identification of manganese nodule rich subareas and the subsequent visual inspection of such areas during a single dive. Producing accurate bathymetric maps for a derivative-based classification of the terrain was a pre-requisite for the mission task.

In this paper, we present methods to fully automate the processing steps to derive accurate bathymetric information from deep diving AUVs or other platforms performing multibeam surveys. A validation dataset is presented that allows comparing different algorithms using an artificially altered high-resolution ship-based dataset with known drift.

## 2. Related Work

Traditionally, the range information from the MBES is recorded by the AUV during the mission. After AUV recovery, the data are downloaded from the vehicle and processed in a semi-manual way using one of the available commercial software packages, free software or self-written procedures. In any case, this involves merging AUV navigation with the sonar range data, removing orientation, translation, and time offsets between different sensors and flagging bad beams manually or using heuristics [12].

Manufactures of acquisition hardware often define specific file formats for their systems and provide or recommend proprietary processing software. As a result, several software solutions exist to deal with different data formats. Processing software includes (but is not limited to) Caris HIPS and SIPS [13] or PDS [14] from Teledyne, QPS sells Qimera [15], HYPACK includes the HYSWEEP module [16], and SONARWIZ [17] also has a bathymetry module, to name a few.

In addition, the open source software package MB-System [18] handles a variety of data formats and is widely used as an alternative for commercial software packages in academia. As part of its functionalities, it provides the mbnavadjust tool [19] to handle unbounded navigation drifts, by dividing survey lines into multiple sub-tracks. Based on the original navigation, two overlapping tiles are presented in the MB-Systems software to an operator, who then manually aligns the maps towards each other by matching contour lines and features. While the UI supports direct manipulation of latitude and longitude, it is possible to change the height translation as well. The software additionally provides the option to automatically calculate solutions by maximizing the cross-correlation between two tiles. The automatic calculation sometimes creates errors and should be supervised by an expert operator. The pairwise solutions are combined into an optimization problem to form a globally consistent solution (Section 3.4).

One alternative solution to the problem is a Kalman filter. While a vehicle model is used to predict the next state, the measured offsets are used as observations for the update step. The following methods share the use of the Kalman filter as a basis, but differ in the way they integrate hydroacoustic measurements as an observation for the filter. Roman et. al. [20,21] generate sub maps of which the sizes are determined by information heuristics. Sub maps are matched pairwise using cross correlation and refined using the iterative closest point (ICP) algorithm in 6DoF. The matches are used as observations for a state extended Kalman filter (EKF). Barkby et al. [22] use a particle filter in addition to an EKF, where, instead of modeling the belief state as a Gaussian approximation, each particle holds a discrete path possibility. Each particle maintains its individual gridded map, including depth estimates alongside uncertainties for each cell. While a parameterized probability function restricts the possible belief states, the particle filter is only limited by the number of particles and can, in principal, represent arbitrary belief functions.

Barkby and Williams [23], using the Gaussian process and predicting the map in unknown regions, were able to employ a particle filter without actual overlap between tracks. Stuckey and Roger [24] used pre-defined matching areas in order to generate observations for the EKF. Whenever a vehicle passes such an area, it can update its navigation based on the previous visits. They evaluated their approach based on ship-based bathymetry alongside simulated data.

This paper details a workflow for matching tile pairs. The results are evaluated using an actual AUV dataset in addition to a very accurate ship-based dataset with RTK corrected GNSS navigation. Into the latter, an artificial drift is introduced. Corrections of this dataset can be evaluated against a ground truth. In contrast to [25], our method has been applied and is derived from actual data, including real artifacts and data noise. Along with our method, we provide an evaluation metric and publish the original and altered dataset so that the performance of different algorithms can be compared quantitatively.

## 3. Tile Matching Workflow

**Figure 1:**
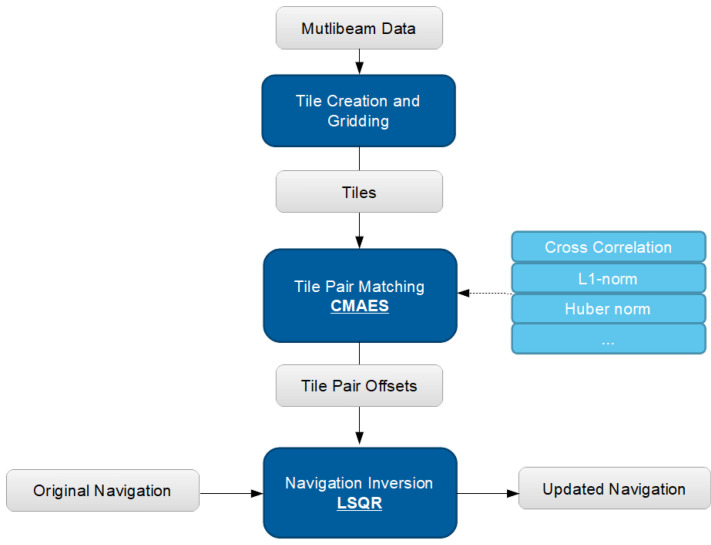
Overview of the Tile matching process. Data objects are shown in gray.

This section details the processing steps necessary to perform the tile matching from raw data to an updated navigation. Figure 1 provides an overview of the workflow.

The first step is to pre-process the raw multibeam data; here, this is achieved using MB-Systems [18]. Static translation offsets in x, y, and z, as well as for rotation (pitch, roll, and yaw) are considered between the common reference point, the motion reference unit and the transducers. In addition, any time delay between multibeam, navigation, and motion data should be resolved. As bottom detection in multibeam systems can be unreliable, the data are flagged for artifacts and noise by applying heuristics for spike and excessive slope detection. Finally, the correct sound velocity profile (SVP) is used for correct ray-tracing. Unflagged soundings are exported alongside the ping and beam number as a 3D point cloud and are used as input data for the proposed algorithm. Up to this point, all of the above-mentioned steps follow a common workflow for any kind of multibeam data processing. We relied entirely on the MB-System and the related commands that are described in the MB-System tutorials and man-pages.

### 3.1. Tile Creation and Gridding

The point cloud data are divided into tiles *T* containing *n* consecutive pings and their respective seafloor soundings across the swath. For each tile, the extreme values for longitude and latitude are determined and define the grid bounds. The cell size *c* defines the grid resolution. The cell values are determined with Gaussian weighting. In contrast to binning, where every sounding is sorted into a specific grid cell, this allows for a higher grid resolution as the cell values are sampled from a continuous function:

(1) $$T\left(x,y\right)=\frac{\sum_{i=1}^{n}w_{i}\left(x,y\right)*z_{i}}{\sum_{i=1}^{n}w_{i}\left(x,y\right)},$$

where $z_{i}$ is the z-value of the *i*th sounding for the given tile.

(2) $$w_{i}\left(x,y\right)=\frac{e^{-\frac{d_{ixy}^{2}}{2\sigma^{2}}}}{\sqrt{2*\pi *\sigma^{2}}}$$

is weighting function with σ=20∗c and $d_{ixy}$ is the euclidean distance between the grid cell at (x,y) and the *i*th sounding. For computational reasons, the weighting factor $w_{i}$ is set to zero for points with a distance further than 2.576σ.

The cumulative weights are stored alongside the final value for each grid cell:

(3) $$W\left(x,y\right)=\sum_{i=1}^{n}w_{i}\left(x,y\right)$$

### 3.2. Objective Function for Optimal Tile Offsets

**Figure 2:**
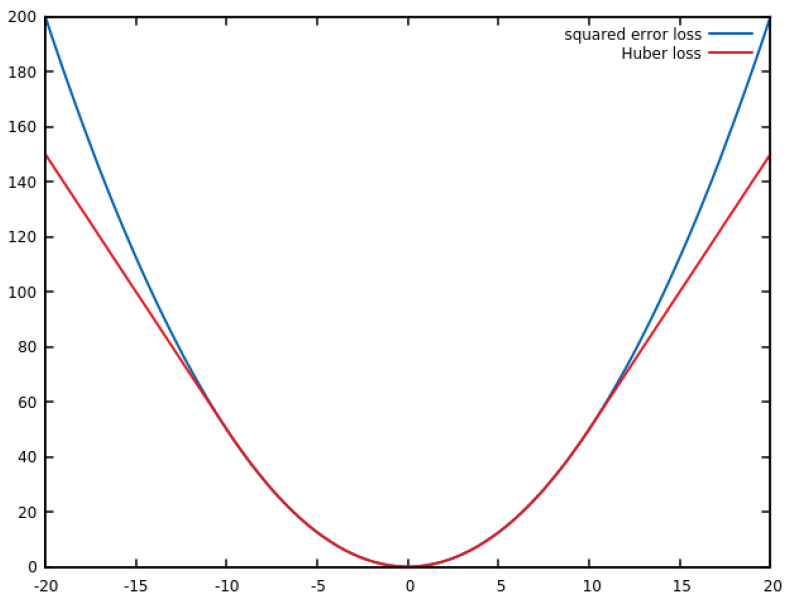
Comparison of the two loss functions, Huber and squared error, for δ=10.

The original navigation is used as a starting point to find tile matches. In order to determine whether two tiles, $T_{1}$ and $T_{2}$, have an initial overlap, the grid bounds are used. For tile pairs with an initial overlap of more than 25%, the optimal offset $o^{*}$ is determined by minimizing the following objective function:

(4) $$o^{*}=\underset{s}{\mathrm{argmin}}f\left(s\right)=\underset{s}{\mathrm{argmin}}\frac{\sum_{\left(x,y\right)\in \left(T_{1}\cap T_{2_{s}}\right)}^{}W\left(x,y,s\right)*L\left(T_{1}\left(x,y\right),T_{2_{s}}\left(x,y\right)\right)}{\sum_{\left(x,y\right)\in \left(T_{1}\cap T_{2_{s}}\right)}W\left(x,y,s\right)}$$

and

(5) $$W\left(x,y,s\right)=\frac{1}{W_{1}\left(x,y\right)*W_{2_{s}}\left(x,y\right)}$$

where $T_{2_{s}}$ and $W_{2_{s}}$ are the second grid and its weights shifted by *s*. Bilinear interpolation is used for subpixel accuracy. The loss function *L* evaluates the value difference between two grid cells. The Huber norm, shown in Figure 2, is used as it combines the advantages of a quadratic error norm with the robustness against outliers of the absolute norm for larger differences:

(6) $$L\left(a,b\right)=\left\{\begin{array}{cc} \frac{1}{2}{\left(a-b\right)}^{2} & for|a-b|\leq \delta \\ \delta |a-b|-\frac{1}{2}\delta^{2} & \;\mathrm{otherwise} \end{array}\right.$$

**Figure 3:**
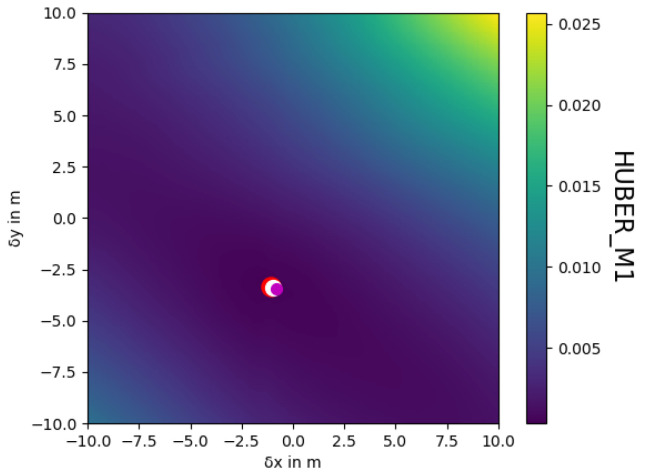
Example objective function for a specific tile pair (See Figure 4). While the center of the plot at 0/0 corresponds to the current and faulty navigation, the three dots represent the local solution (white), the globally consistent solution (purple), and the ground truth (red).

**Figure 4:**
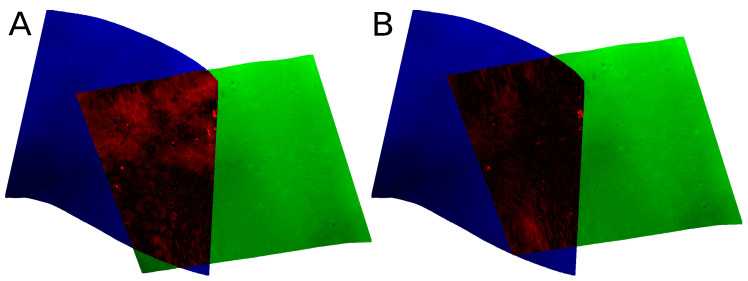
Example of a tile pair before (**A**) and after (**B**) navigation correction. Each image shows a tile pair with the heights color-coded in blue and green, respectively. The overlapping area shows the absolute difference between both tiles from black to red. These shifts are represented in Figure 3 as the center of the plot and the blue circle, respectively.

If there is no overlap for a given shift, the value of the objective function is set to the maximum value possible. In our method, the z dimension is not considered, as pressure sensors have bounded errors and are, therefore, not prone to drifting. As an example, Figure 3 visualizes the function for the tile pair shown in Figure 4.

### 3.3. Optimization Using CMAES

The resulting optimization function is non-convex and can have multiple local optima. In order to solve this, the covariance matrix adaptation evolution strategy (CMAES) [26] is employed. In each iteration step, a new generation is sampled from a multivariate normal distribution over the space of possible shifts. The samples are recombined according to their respective objective function values by selecting the best *n* samples and forming a new mean value for the distribution. Mutation is achieved by calculating a new covariance matrix that increases the likelihood of previously successful search steps.

The overlap between two tiles is changing alongside its shift. The objective function is normalized over the area to avoid bias towards small overlaps. The correct solution is potentially outside the considered search area with no overlap at all. It is impossible to determine a match in such a case; however, a minimum for the objective function exists. We consider a match valid if the overlap is larger than a fixed number of cells $|T_{1}\cap T_{2_{o^{*}}}|\geq n_{min}$ or if the overlap ratio between tiles is larger than $n_{p}$ and the overall objective function is smaller than a given threshold $f^{*}<f_{max}$. If both conditions hold true, we consider the offset to be correct.

### 3.4. Navigation Correction

The individual tile pair offsets are combined into a global consistent navigation by solving a sparse over-determined least square matrix problem of the form AX=B. The same method is used in MB-Systems mbnavadjust [19], which is described below.

The solution vector *X* models the absolute offset in *x* and *y* for the center of each of the *m* tiles:

(7) $$X={\left(x_{1},y_{1},\ldots ,x_{i},y_{i},\ldots ,x_{m},y_{m}\right)}^{T}$$

The solution vector size is therefore twice the number of tiles: (2m×1). The constraint matrix *A* models two kinds of constraints: the relative offset between tile pairs and a term that penalizes the first derivative of the navigation adjustment. The first constraint includes all valid tile matches, where each match corresponds to one row vector in *A*. The vector contains −1 for columns related to the first tile *a* and 1 for columns related to the second tile *b*. All other column values are set to zero. The offset value $o_{a,b}$ corresponds to a value in *B*:

(8) $$-x_{a}+x_{b}=o_{a,b}\left[0\right]$$

(9) $$-y_{a}+y_{b}=o_{a,b}\left[1\right]$$

The second constraint minimizes the derivative of the adjustment by considering the consecutive tiles *i* and *j*:

(10) $$\frac{-x_{i}+x_{j}}{-T_{i}+T_{j}}=0$$

(11) $$\frac{-y_{i}+y_{j}}{-T_{i}+T_{j}}=0$$

where $T_{i}$ is the timestamps in seconds of tile *i*. Again, the corresponding column values in *B* are set to $\frac{-s}{-T_{i}+T_{j}}$ for tile *i* and $\frac{s}{-T_{i}+T_{j}}$ for tile *j*. The smoothness term *s* determines how strictly the solution needs to fit individual offsets in comparison to how rough the navigation changes are. The larger this value, the smoother the corresponding solution at the price of less accurate offsets between tiles. Because the second constraint type adds 2(m−1) constraints, the total number of constraints for more than one match is always larger than the number of unknowns. The system is therefore over-determined and can be solved using the sparse equations and least squares (LSQR) algorithm [27].

In a final step, the navigation offsets are applied to the multibeam data based on the timestamps. For the center ping in the time of each tile, we directly apply the found solutions. Linear interpolation in time is used to find the correction for pings in between.

## 4. Evaluation

The strategy to evaluate the algorithm is two-fold. We will use a dataset recorded by an AUV and apply the algorithm to eliminate natural occurring drifts (Section 4.5). We can evaluate the results based on criteria such as variance of the resulting bathymetric grid by visually inspecting and checking the map for correct matches or by comparing the corrected navigation to dead reckoning navigation with USBL or LBL fixes. This last approach is used in the literature to evaluate the performance of algorithms [20,21]. However, this does not allow direct comparisons between different parameter values or algorithms. Section 4.2 introduces a method that allows adding a known drift to an existing navigation. The drift is added to a very precise ship-based navigation. The sonar data are recalculated accordingly and the original data are used as ground truth.

### 4.1. Ship-Based Data Set

**Figure 5:**
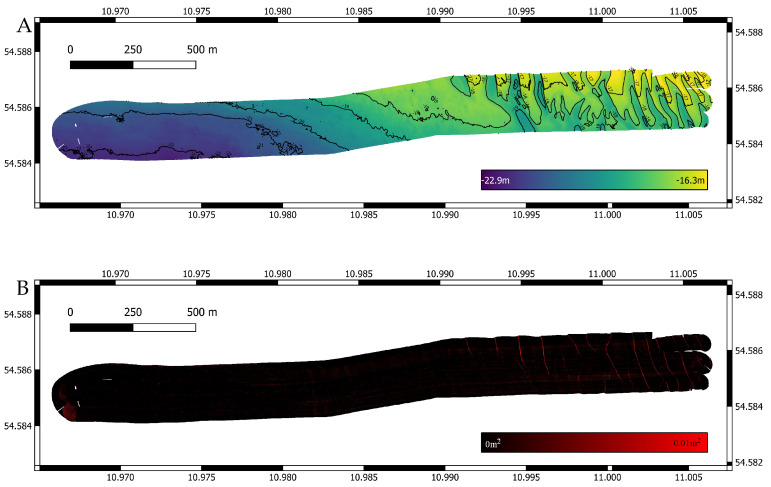
Survey with original navigation. (**A**) Bathymetric map with a cell size of 0.5 m. (**B**) Variance for the same grid cells. As expected the variance is reasonably low. In certain areas, a minimal increase can be observed, which is due to slightly mismatched heights between individual tracks. A navigation drift is not the cause, which can be further validated by a visual inspection of features like rocks, which match perfectly.

This dataset [28] was recorded with the research vessel Littorina at Fehmarn Belt in the western part of the Baltic Sea [29]. The multibeam used was the RESON SeaBat T50 Extended Range (Table 1). With 600 beams and a swath opening angle of 120${}^{\circ }$, the seafloor in an approximate 20 m water depth was recorded. The navigation was recorded using two Septentrio differential global positioning system (DPGS) antennas. Real-time kinematic (RTK) was used to enhance the position data, which resulted in centimeter-level accuracy. Figure 5 shows the bathymetry and variance for this dataset.

This dataset was processed manually using Qimera to account for time delays, static offset, and sound velocity corrections, as well as flagging of erroneous soundings.

An interesting feature of this particular area are large sub-aqueous dunes [30] of up to 2.35 m in height. These dunes define the morphology and are perfect targets for evaluating terrain-based navigation algorithms.

### 4.2. Drift Model

We consider the navigation *N* to be an ordered list of 3-tuples $<t_{i}x_{i}y_{i}>$, where *t* is the timestamp and *x* and *y* are the corresponding projected coordinates $\vec{n_{i}}$ in a Cartesian coordinate system (in this case, UTM zone 32). The *z*-dimension and orientation can be ignored, as we are only interested in a drift on the *x*-*y*-plane.

We introduce an additional drift $\vec{d_{i}}$ to each navigation point by sampling a random acceleration $\vec{a_{i}}$ between navigation points from a Gaussian distribution with $\Sigma =\left[\begin{array}{cc} \sigma^{2} & 0\\ 0 & \sigma^{2} \end{array}\right]$ and σ=0.0001 [m/s^2^]:

(12) $$\vec{a_{i}}∼N_{2}\left(\vec{0},\Sigma \right)$$

and calculate the drift and updated navigation $N^{\prime }$ accordingly:

(13) $$\delta t_{i}=t_{i}-t_{i-1}$$

(14) $$\vec{v_{i}}=\vec{v_{i-1}}+\delta t_{i}*\vec{a_{i}}$$

(15) $$\vec{d_{i}}=\vec{d_{i-1}}+\delta t_{i}\vec{v_{i-1}}+0.5\delta t_{i}^{2}\vec{a_{i}}$$

(16) $$\vec{n_{i}^{\prime }}=\vec{n_{i}}+\vec{d_{i}}$$

**Figure 6:**
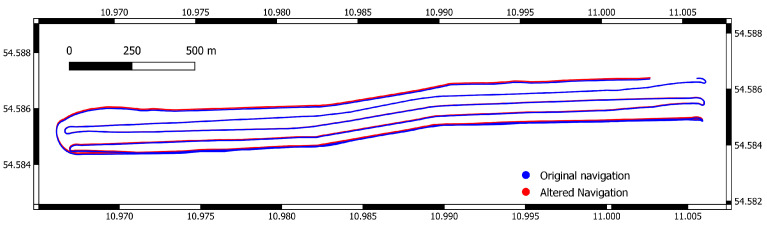
Original and altered navigation. In the beginning of the survey, original and altered navigation are still aligned, while the difference is most prominent near the end. Coordinates are in UTM (zone 32N).

The altered navigation of the ship-based dataset is used as a new navigation for the multibeam data (See Figure 6).

### 4.3. Navigation Evaluation Metric

To create a benchmark, a similar approach to the evaluation metric for traditional visual SLAM-algorithms [31] was used. The goal was to compare a renavigated solution with a known original navigation, which translates to the problem of comparing trajectories. As long as a solution to the renavigation problem satisfies all relative constraints given by the data, we consider it to be valid. Therefore, a global translation between ground truth trajectory and renavigated solution has to be allowed. For each trajectory, a mean value is calculated. The difference is used to align them. The average euclidean distance between points with the same timestamp is the evaluation metric.

### 4.4. Results

The tile matching is first applied to the ship-based dataset with added simulated drift. Table 2 shows the parameter settings that are common for all experiments. The parameter values were manually chosen to achieve good results for another survey with the same hardware setup. While decreasing *c* generally leads to better results, it negatively impacts the run time of the algorithm. It should be chosen in relation to the beam footprint of the multibeam system, and is therefore dependent on the altitude above ground. σ should be a multiple of *c*. Since multibeam missions tend to have a fixed overlap between tracks it is possible to choose $n_{p}$ accordingly. With a 50% overlap in the mission setup, a value of 0.25 will allow for a significant amount of matches even if the tile bound are not aligned across tracks. Section 3.2 introduced the Huber norm as a loss function for evaluating height differences between overlapping pixels. It is tested against negative cross correlation, the truncated quadratic function and the L1 norm. Parameters have to be adjusted according to the used objective functions (see Table 3).

**Figure 7:**
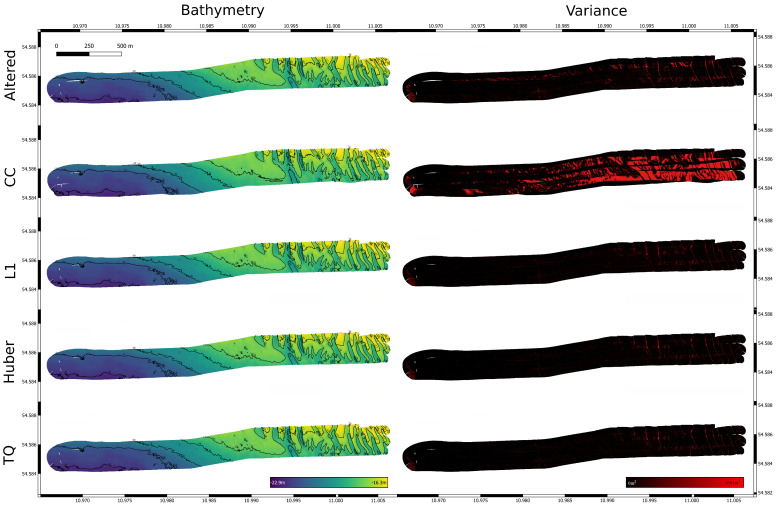
Survey with altered and readjusted navigation for trial 1 using different norms: cross correlation, L1, Huber and truncated quadratic.

**Figure 8:**
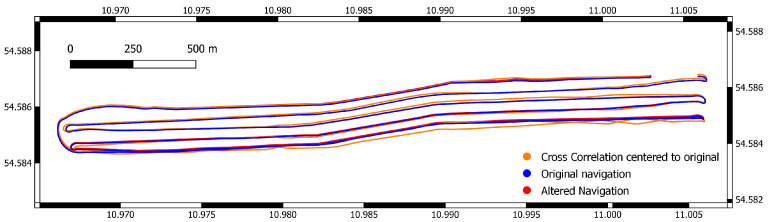
Original and altered navigation alongside the renavigated solution centered towards the original navigation using cross correlation.

Figure 7 shows the resulting variance for the altered dataset and the corrected versions. While L1, Huber, and truncated quadratic loss functions significantly improve the resulting bathymetry, this is not true for cross correlation. The direct implementation of this loss function into our algorithm favors overlap with larger depth values in general, so while locally this approach might be feasible, the global search for a solution using CMAES results in incorrect solutions (see Figure 8), this is reflected in the navigation distance metric. Table 4 shows the increased distance for cross correlation, while it is significantly reduced for all other loss functions.

**Figure 9:**
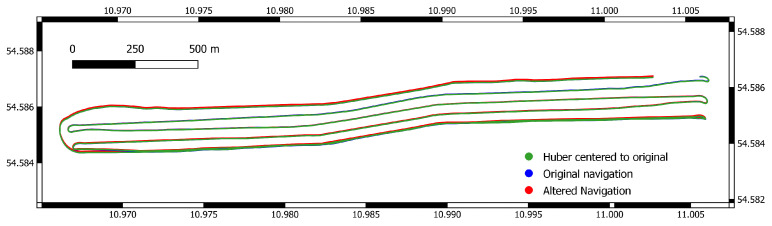
Original and altered navigation alongside the renavigated solution centered towards the original navigation using Huber loss for trial 1.

The norms Huber, L1, and truncated quadratic perform much better. This dataset was cleaned manually; therefore, no large spikes exist in the data, the difference between the three approaches is minimal. Huber and the truncated quadratic norm are designed to work with noisy data, for uncleaned data a relative increase in performance can be expected. The resulting navigation for all three norms are generally quite similar (see Figure 9). Over all trials, these norms lead to a better solution closer to the ground truth, except for trail 8. Here, the altered navigation leads to a significant drift between the first and last line. While normally they are sufficiently close to allow loop closing, here, this is not the case. Instead one wrong match is found, which negatively effects the solution.

**Figure 10:**
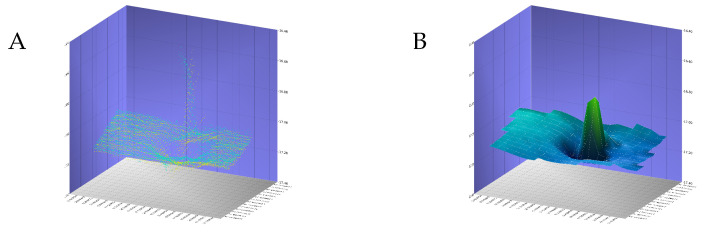
Section of the point cloud data visualized in Qimera after the navigation correction using Huber loss as individual sounding color-coded by file (**A**) and surface (**B**). The height is exaggerated to make the feature more prominent. Instead of two halves of one object, the algorithm directly matches the halves to be exactly at the same position. This bias can be observed multiple times in the survey data.

For the remaining trials, the greatest variance was observed in areas where the original solution ws inconsistent, too. Manual inspection confirms that there is no trivial better solution for these problematic areas, since the increase is not due to a drift in the navigation. Another interesting observation was made: when objects on the ground are ensonified with an angle, the point cloud data show shadow effects in the form of lacking soundings behind the object. Our algorithm tends to directly align the visible areas with each other, ignoring the unknown shadow regions. If the shadow area is on different sides of the object for a tile pair, a small bias in the form of a consistent offset towards one another is introduced Figure 10 visualizes this effect.

### 4.5. AUV Data Set Results

The AUV dataset was recorded in 2015 during RV SONNE cruise SO242 with GEOMAR AUV Abyss [32] using a RESON SeaBat 7125 MBES (200 kHz). The working area was the DISCOL experimental area (DEA) in the Peru Basin [33], where Mn-nodules cover the seafloor. A total of 122,619 pings were recorded.

In contrast to the ship-based dataset, this dataset was processed exclusively automatically. Merely heading, pitch, and roll bias were fixed beforehand. Data cleaning using heuristics and correction for time lags in the data were conducted using MB-System in script form.

**Figure 11:**
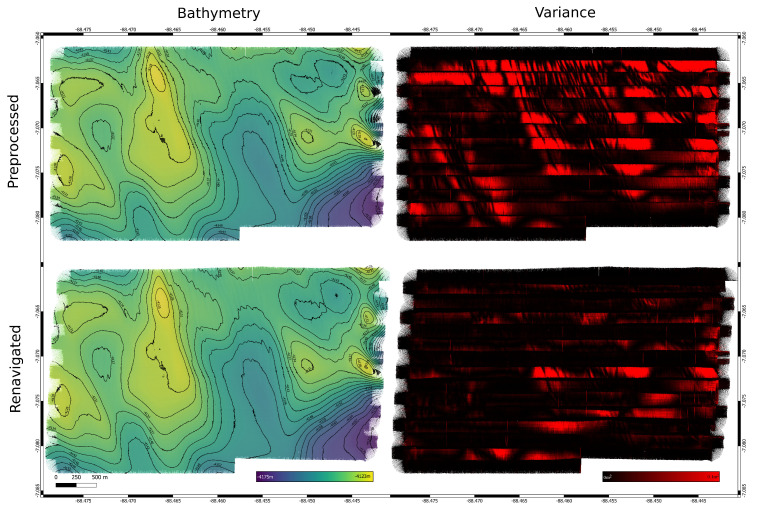
Bathymetry and variance for a 2 m resolution grid before and after navigation correction. Artifacts in the form of “waves” are clearly visible, despite an effort to reduce biases in the data as good as possible. The original navigation leads to overlapping tracks mismatching in height. Naturally this is visible in the variance, too. Applying the corrected navigation reduces these artifacts significantly.

**Figure 12:**
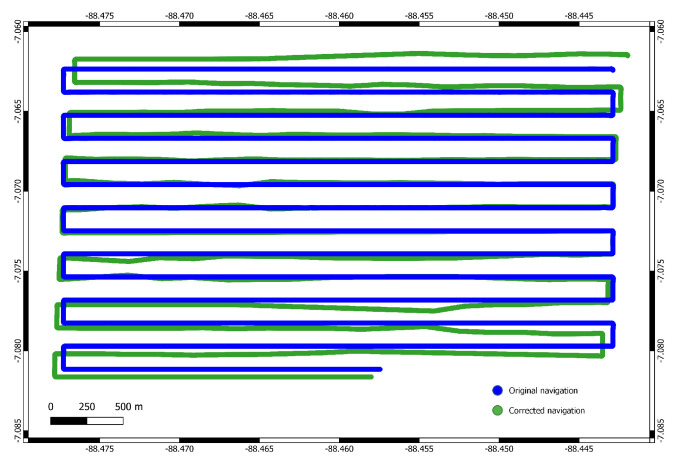
Original navigation and corrected navigation. Naturally the original navigation solution is much more closer to the planned mission, a regular lawn-mowing pattern, the corrected solution shows some significant drifts.

The parameter settings are given in Table 5. Results of the pre-processed and renavigated dataset using the Huber norm are shown in Figure 11. While the renavigation produced a very good fit in the northern region, there are still some artifacts just east of the map center. This artifact is present in the original navigation, but the algorithm fails to find a better match for this specific track. The reason for this is that $f_{max}$ was deliberately set conservatively. As a result, the found matches were discarded as invalid. The solution was not corrected in this area, but also no wrong correction was introduced. As shown by the variance, the algorithm was overall able to correct the navigation and the quality of the bathymetric map was improved. The navigation solution alongside the original is shown in Figure 12. The total accumulated drift for the survey is larger then 200 m. In total, the algorithms runtime was 8.5 min compared to a mission time of 10 h.

## 5. Conclusions

We presented an automated algorithm to correct navigational drifts by aligning multibeam data. By applying drift corrections only on the 2D plane (x and y dimensions), it was shown that this algorithm improves the navigation for AUV data. Our method can handle time gaps in the data, which allows to remove tracks with poor quality beforehand. The knowledge of a vehicle model is not necessary as the algorithm purely relies on the original navigation and the bathymetric dataset. Since detailed information about the platform is not always available, this is a significant advantage.

In an effort to allow for an objective and quantifiable evaluation of algorithms and their settings, we created a benchmark dataset of a ship-based multibeam survey modulated with an artificial, but known drift.

In the future, we plan to extend the algorithm to encompass height differences (z dimension) and rotations. This will be reflected in the benchmark dataset as well. Adding additional surveys and altered navigations to the benchmark will allow for even more accurate validations of algorithms and parameter settings.

## Figures and Tables

**Table 1:** Specification RESON T50 extended range.

Frequency	400 kHz
Across-track beam width	0.5${}^{\circ }$
Along-track beam width	0.5${}^{\circ }$
Number of beams	10–1024
Swath angle	10–165${}^{\circ }$

**Table 2:** Shared parameter settings for the vessel dataset experiment.

Parameter	Value
Number of swaths/pings per Tile *n*	500
Grid cell size *c*	0.05 [m]
Gaussian weighting σ	0.25 [m]
CMAES initialization $\sigma_{CMAES}$	0.5 [m]
Minimum overlap ratio $n_{p}$	0.25

**Table 3:** Maximum objective function value $f_{max}$ for different loss functions. For none-normalized cross correlation (*), the objective function value is directly related to the depth of the soundings. It is therefore not feasible to select a meaningful value as a threshold and it is not considered for the quality of a match at all.

Loss Function	$f_{\max}$
(negative) Cross Correlation	0 *
L1	0.02
Huber	0.0005
Truncated Quadratic	0.0005

**Table 4:** Average Euclidean distance of ground truth navigation to altered navigation and renavigated solutions for different norms based on nine different altered navigations. The values are rounded to two places after the decimal separator.

Norm	Average Euclidean Distance Metric [m] for Trials									μ	*s*
	**1**	**2**	**3**	**4**	**5**	**6**	**7**	**8**	**9**		
Altered navigation	2.05	1.93	3.61	1.86	1.81	3.50	3.35	3.87	2.29	2.70	0.86
Cross Correlation	9.30	12.48	18.59	9.62	10.24	10.19	12.89	13.63	12.80	12.19	2.89
L1	0.93	0.94	0.72	0.84	0.96	2.22	1.09	4.34	0.84	1.43	1.18
Huber	0.76	1.08	0.88	1.42	0.85	2.11	1.18	4.54	0.72	1.51	1.22
Truncated Quadratic	0.76	1.08	0.87	1.03	0.96	1.71	1.19	4.55	0.72	1.43	1.20

**Table 5:** Parameter settings for AUV dataset experiment.

Parameter	Value
Number of swaths per Tile *n*	500
Grid cell size *c*	0.5 [m]
Gaussian weighting σ	0.75 [m]
CMAES initialization $\sigma_{CMAES}$	5 [m]
Minimum overlap ratio $n_{p}$	0.15
maximum objective function value $f_{max}$	0.1

## Data Availability

The benchmark dataset [28] is available here: https://doi.pangaea.de/10.1594/PANGAEA.938637.

## Abbreviations

The following abbreviations are used in this manuscript:

AUV	autonomous underwater vehicle
MBES	multibeam echosounder system
GNSS	global navigation satellite system
RTK	real time kinematic
IMU	inertial measurement unit
INS	inertial navigation system
USBL	ultra short baseline
LBL	long baseline system
ICP	iterative closest point
EKF	extended Kalman filter
SVP	sound velocity profile
CMA-ES	covariance matrix adaptation evolution strategy
LSQR	spare equations and least squares
DPGS	differential global positioning system
DEA	DISCOL experimental area
